# Role of Vitamin C in Prophylaxis and Treatment of Gout—A Literature Review

**DOI:** 10.3390/nu13020701

**Published:** 2021-02-22

**Authors:** Olga Brzezińska, Filip Styrzyński, Joanna Makowska, Konrad Walczak

**Affiliations:** 1 Department of Rheumatology with Subdepartment of Internal Medicine, Medical University of Lodz, 90-549 Lodz, Poland; styrzynski@gmail.com (F.S.); joanna.makowska@umed.lodz.pl (J.M.); 2 Department of Internal Medicine and Nephrodiabetology, Medical University of Lodz, 90-549 Lodz, Poland; konrad.walczak@skwam.lodz.pl

**Keywords:** Vitamin C, ascorbic acid, nutrition, gout, hyperuricemia, diet

## Abstract

Gout, known as “the disease of the kings”, is the most frequent type of arthritis. It results from sustained hyperuricemia that leads to monosodium urate crystal deposition in joint structures and soft tissue. Environmental factors such as diet affect the incidence of gout; there is a known relationship between the occurrence of an acute attack of gout and the consumption of alcohol and meat; and a low purine diet is a widely recognized nonpharmacological method of supplementing the treatment and preventing recurrence of arthritis. This review aims to summarize the current knowledge about the role of vitamin C in prevention and treatment of gout. A PubMed/Medline database search on the role of vitamin C in purine metabolism was done. Reports from in vitro and animal studies seem to be promising and to allow explanation of the physiological relationship between vitamin C and uric acid. Most epidemiological studies indicate a significant correlation between high vitamin C intake and lower serum uric acid levels. Despite promising observations, there are few observational and interventional studies, and their results do not clearly define the benefits of a high daily intake of vitamin C in preventing the development and recurrence of gout.

## 1. Introduction

Vitamin C (VC), known as ascorbic acid, plays a pleiotropic function in organisms. Its role in regulation of various biological processes has been widely investigated since its discovery in 1932 by Albert Szent-Györgyi, a Hungarian biochemist [1,2]. Despite being essential for the homeostasis, primates, such as flying mammals and guinea pigs, have lost their ability of endogenous production of ascorbic acid over their centuries of evolutional changes. This molecular mechanism is explained by the lack of l-gulonolactone oxidase, which catalyzes the last step of VC biosynthesis [3]. Therefore, inadequate supplementation leads to the health conditions caused by VC deficiency. The most known example of the disease is scurvy (latin: *scorbutus*), from which the official name of ascorbic acid originated (*a-scorbic*, thus *against scurvy*), which was found amongst sailors in the 18th century [4]. However, it was first Hippocrates who described a similar clinical presentation termed “ileos ematitis”—“the mouth feels bad; the gums are detached from the teeth; blood runs from the nostrils … ulcerations on the legs; some of these heal … skin is thin.” [5]. Current data provide us with increasing knowledge about the possible influence of vitamin C on prevention and treatment of, e.g., cardiovascular diseases, cancer, inflammatory diseases spectrum, as well as hematopoietic and soft tissue disorders [6,7,8]. The most prominent physiological process in which VC is involved is antioxidation. However, pharmacological features of vitamin C might be influenced by several factors, including genetics, environment, and individual lifestyle approach. It remains unclear whether the observations on ascorbic acid supplementation have a correlative or causative relationship with outcomes, whether the optimal condition-related dose of VC could be estimated, what source of vitamin C is required, and how to implement it in a dietary or clinical approach [9,10].

Hyperuricemia and, consequently, gout—historically, called “the disease of the kings” due to disproportional affliction amongst royalties—is one of the alarming medical conditions of modern and wealthy societies [11]. Nowadays gout is perceived as a systemic disease affecting more than just the musculoskeletal system [12,13]. The prevalence of hyperuricemia and gout is increasing worldwide, especially in high-income and economically developing countries. Epidemiological data are incomplete, but available analysis gives information about a prevalence of circa 20% for hyperuricemia in adults [14,15], 14% for hyperuricemia in adolescents [16], and 2.6% for gout [17]. It means that almost every fifth person is affected by increased uric acid level and its consequences.

The development of gout is currently considered on a continuum, starting from asymptomatic hyperuricemia, to urate-crystal deposition in joint cavities, and finally gout and its complications [18,19]. The risk of reaching the final state is increased in the presence of persisting hyperuricemia, and the correlation is linear to exponential [20]. Elevated level of uric acid is therefore the key factor for the identification of treatment opportunities. 

Evolutionally, humans have lost the ability to produce uricase, an enzyme responsible for the metabolism of uric acid and its transformation into a more soluble one—allantoin [21,22,23,24,25,26]. On the other hand, uric acid serves as a strong antioxidant and reduces the risk of oxidative stress in the human organism [27]. Some authors underline the possible complementary function of uric acid and vitamin C as antioxidants, supported by evolutional loss of ability to synthesize endogenous vitamin C and to metabolize uric acid [28].

Although dietary approach seems to play a main role either as an effective preventive measure or in provoking gout attacks, a thorough understanding of the interactions between uric acid and vitamin C in the human organism remains unsatisfactory [29,30]. Several studies have found significant correlations but without identifying their causal relationship [31]. Considering the possible mechanism of the uric acid-lowering properties of vitamin C, the uricosuric effect seems to outweigh others. However, most recent studies also suggest another important role of ascorbic acid in the reduction of urate-induced inflammation by inhibiting activation of responsible NLRP3 inflammasomes. Despite promising results, this novel approach requires further evaluation [32,33].

This review aims to summarize the current knowledge on the role of VC in the prevention and management of hyperuricemia and gout. The actual understanding of its molecular role in disease development as well as potential clinical benefits of ascorbic acid supplementation are presented. 

## 2. Materials and Methods

In order to obtain comprehensive knowledge of the role of vitamin C in the metabolism of purines, its effect on the concentration of uric acid in the serum, and its role in the prevention and treatment of gout, this work includes molecular studies as well as observational and intervention studies with humans. The review includes scientific articles available in the PubMed/Medline database published by 24 November 2020. 

**Figure 1:**
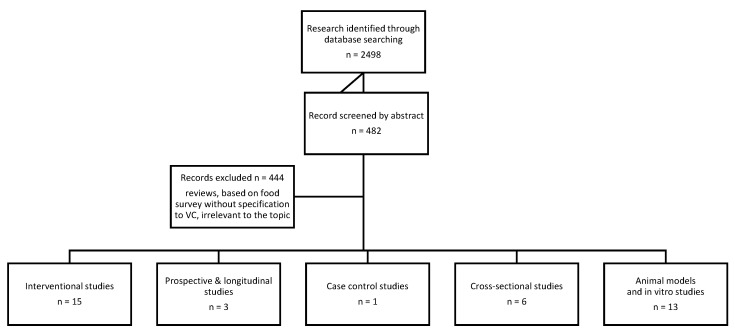
PRISMA (Preferred Reporting Items for Systematic Reviews and Meta-Analyses) flowchart showing the useful study selection process. *n*: number, VC: vitamin C.

Two groups of words were used as search criteria. The first search concerned vitamin C: “vitamin c”, “ascorbic acid”, “antioxidants”. The second search related to uric acid metabolism: “gout” or “hyperuricemia” or “purine metabolism” or “monosodium urate crystals”. The key words of both groups were joined together via the “AND” hyphen in all available configurations. No exclusion criteria were employed. The search results were checked for relevance, and 38 articles were selected for the final review. The article selection process is presented in Figure 1. 

This article presents the results of molecular studies as well as observational and intervention population studies, with the results being discussed in line of increasing clinical credibility.

## 3. Results

### 3.1. Molecular Function of Vitamin C in Gout Etiopathogenesis

Pleiotropic effects of the vitamin C have been widely researched over decades. Amongst its physiological mechanisms, antioxidant and cofactor functions seem to play an important role in maintaining homeostasis. Plasma devoid of ascorbate is extremely susceptible to oxidant stress and peroxidative damage, despite the presence of another endogenous antioxidant [1,10]. The inability of endogenous production of vitamin C, e.g., in humans and guinea pigs, resulting from evolutional mutations in the L-gulonolactone oxidase gene, leads to the need for exogenous supplementation [34,35].

Uric acid is known for its detrimental influence concerning the clinical consequences of hyperuricemia and gout [36]. On the other hand, its high antioxidative properties need to be outlined and recognized in terms of homeostasis [27,37,38]. Low levels of uric acid are linked to progression of nervous system degeneration [39,40,41,42]. Certain studies suggest that a loss of the active uricase gene in humans might have been another evolutional adaptation that counteracts vitamin C deficiency by increasing serum uric acid levels. In other species and non-primate mammals, the uricase enzyme is responsible for the degradation of uric acid into soluble compound allantoin [21,22,23,24,25,26].

The relationship between vitamin C and uric acid levels is considered relevant according to both a genetical and an environmental approach. In a large randomized study of 106,147 individuals, an association of high plasma vitamin C and low plasma urate (consequently low risk of hyperuricemia) was confirmed. However, no causal genetic connection was found. The SLC23A1 gene variant linked to high plasma vitamin C did not result in different plasma urate levels or a lower risk of developing hyperuricemia [31]. On the other hand, a synergistic interaction between vitamin C levels and an elevated genetic risk score for hyperuricemia has been suggested amongst African-American men [43].

**Figure 2:**
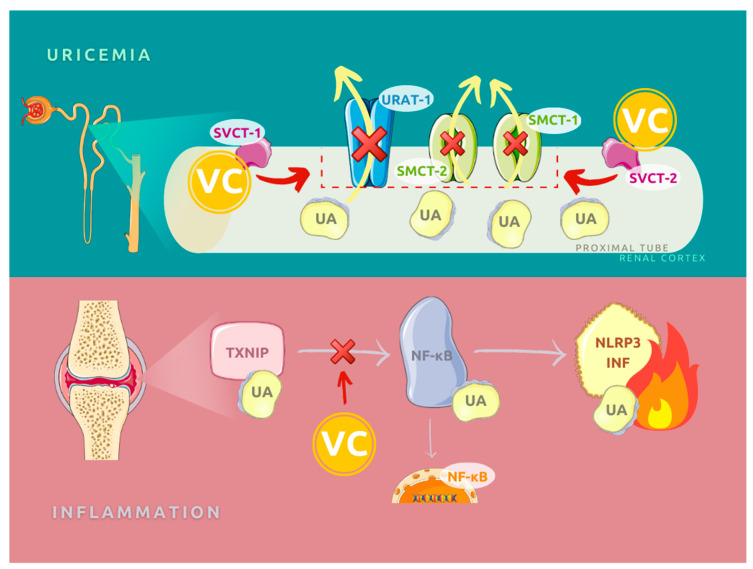
Molecular role of vitamin C in the transport of uric acid in the proximal renal tubules and in inflammatory process regulation. VC: vitamin C, UA: uric acid, URAT-1: urate transporter 1, SMCT: sodium-coupled monocarboxylate transporter, SVCT: sodium-dependent vitamin C transporter, TXNIP: thioredoxin-interacting protein, NF-κB: nuclear factor kappa-light-chain-enhancer of activated B cells, NLRP3 INF: NLR family pyrin domain containing 3 inflammasome.

Pharmacologically, the uricosuric effects of vitamin C seem to have the highest impact on the hyperuricemia and gout in humans [44,45,46,47]. The possible explanation for this feature relates to the renal tubular reabsorptive transport of uric acid, whereas the increase in secretion remains inconsistent. The molecular mechanism includes involvement of the urate anion exchanger URAT1 (encoded by SLC22A12, responsible for 50% of reabsorption) and two sodium-dependent anion cotransporters of the SLC5 gene family (SLC5A8/SMCT1, SLC5A12/SMTC2) in the proximal tubule [48,49,50]. Although the last two are not urate transporters, research conducted on mice proved a functional coupling between urate and lactate reabsorption in the kidneys concerning SLC5A8 and SLC5A12 co-transporters [51]. Available data suggest that vitamin C could influence the activity of URAT1 and its cofactors by two vitamin C transporters (SLC23A1/SVCT1 and SLC23A2/SVCT2) located also in the proximal tubular epithelial cells [45,52]. See Figure 2.

Current research sheds new light on another mechanism of urate-induced inflammation connected with the role of thioredoxin-interacting protein (TXNIP) in nuclear factor-κB (NF-κB) signaling and its interaction with NOD-like receptor protein 3 (NLRP3). It results in the activation of the NLRP3 inflammasome. TXNIP protein and eventually urate-induced inflammation can be successfully inhibited by vitamin C as an antioxidant [32,33].

### 3.2. Cross-Sectional Studies 

After reviewing the literature, six cross-sectional studies were identified (Table 1). Such research does not allow for sequence of events analysis; however, due to the short time of implementation, the possibility of including a large group of patients allows for the identification of potential connections, whose presence should be confirmed in further long-term observational and interventional studies. 

The identified cross-sectional studies described seven cohorts and almost 61,000 cases. Most of the presented studies included data collected after 2005 [53,54,55,56], but the oldest reported data were collected in the 1990s [57]. Three of the described groups came from South Korea [53,54,56], one each from China [55], the US [58], Australia, and Norway [57]; no African, Eastern, Western and Southern European, Central and South American and Middle Eastern countries were represented. All the presented studies included both women and men over 19 years old, the average age ranged from 47–62, and all authors also defined hyperuricemia as a serum uric acid (SUA) concentration exceeding 6 mg/dL in women and 7 mg/dL in men. Although the systems used to perform the laboratory tests differed between research, the enzymatic method using uricase was proposed for the determination of serum UA (uric acid) concentration in all studies. 

In the presented study, daily vitamin C intake was assessed using two different methods—direct daily recall ongoing from 1 to 3 days [54,55,56] or a food frequency questionnaire (FFQ) containing 80–158 items [53,57,58]. FFQ forms are a standardized tool for semi-quantitative assessment of individual product consumption, such as grains, fish, dairy products, or drinks, adapted to local dietary habits and the need for research on the level of detail obtained. There are over 200 different forms available in literature, ranging in length from 5 to 350 items [59]. It seems that the information obtained on the basis of a FFQ is a bit more complete than one based on direct daily records because they describe dietary habits over the last months, but they may be burdened with measurement error and recall bias.

Most of the presented studies reported higher mean concentrations of SUA in men than in women [53,55,57] and more frequent occurrence of hyperuricemia [54,55]; the others did not use a division according to gender. Previous studies have also reported sex-related differences in SUA. Such a difference between genders is probably due to the estrogen, which purportedly increases urinary excretion of uric acid [60]. There were significant differences between studies in the average level of vitamin C intake in the diet too—the lowest values were found in the Ryu et al. study [54]. The difference was evident even in the results for a Korean population, where the only difference between average VC consumption in each gender group and hyperuricemia and non-hyperuricemia was as high as 20 mg/day. This difference may result from changes in nutritional trends over time, as well as from the characteristics of the population that qualified for each study. The highest values of daily VC consumption were recorded in the US population, and they were more than twice as high as in the Korean cohort of Ryu et al. [54]. Korean authors suggest that this difference may be due to different patterns of vitamin supplementation and its frequency in individual populations. Koreans prefer to use multidisciplinary supplements, such as multivitamins, instead of single-ingredient supplements such as vitamin C and vitamin D [61].

Although hyperuricemia is inherently associated with an increasing risk of gout, only some authors considered the disease to be a significant clinical variable [57]. In a study by Zheng et al. [58], cases treated with preparations lowering UA serum concentration, e.g., allopurinol or febuxostat, were excluded from the cohort. A study designed by Zykava et al. [57] included data from two separate cohorts, one from Australia (Australian Diabetes, Obesity and Lifestyle Study; AusDiab), where 9734 participants were included, and one from Norway (Tromsø Study) with 3031 participants. The two data sets were handled separately because of differences in survey dates, distribution of food intake, and local dietary habits; both groups were of Caucasian origin. Patients suffering from gout were identified either based on a questionnaire, the positive answer to the question “Have you ever suffered from gout?” in the AusDiab Study, or in the self-reported use of allopurinol, tisopurine, febuxostat, etc. in the Tromsø Study. Patients qualified as gout-positive were a separate group, but there was no direct data on their vitamin C intake. The history of clinically evident gout in both cases was treated as a corrective variable similar to body mass index (BMI), estimated glomerular filtration rate (eGFR), hypertension, diabetes, alcohol consumption, and increased physical activity. Higher VC intake (highest quartile) was found to be associated with lower SUA concentration in the female but not the male Australian population; no relationship was found for the Norwegian cohort. This result was not repeated in other studies, where the relationship between VC consumption and the level of UA in raw materials was analyzed based on the division into quartiles. All the Sun et al., So et al., and Bae at al. studies [53,55,56] observed a linear negative association between daily VC dietary intake and SUA. The lack of an observed relationship in the Norwegian and Australian cohorts is difficult to verify because there is no information on the average amounts of vitamin C consumed by the individual groups, and there was no tile-based analysis. Only in the study by So et al. [56] was there no significant difference in the daily consumption of VC between people with hyperuricemia and normouricemia; such a relationship was observed in the other cohorts [53,54,55].

The results of the presented research seem to suggest the existence of a significant relationship between the level of SUA and the daily intake of VC. However, it should be noted that the available studies concerned the populations of only five countries, and even within the same country, the obtained results were not identical, despite similar eating habits and lifestyle of the described cohorts’ members. Due to the significant differences in the daily consumption of individual nutrients between communities and the lack of a clear answer as to which VC values may have a positive effect on the SUA level, it seems advisable to conduct further research on a larger scale. In addition, cross-sectional studies should always be treated as an epidemiological study; they do not provide the possibility of establishing a cause-and-effect relationship between the observed phenomena; therefore, it is necessary to verify their results using long-term, prospective observational studies and interventional studies.

**Table 1:** Cross-sectional studies discussing the association between vitamin C diet intake and hyperuricemia prevalence.

Study	Study Population	Dietary Assessment	Exclusion Criteria	Results
Bae et al. 2014 South Korea [53]	9400 subjects: 3564 males 62.5 ± 9.6 years old 5836 females 61.6 ± 9.8 years old	103-item food frequency questionnaire (FFQ)	Subjects with missing data, total energy intake <500, or >4000 kcal/day; ≥10 missing food items; or missing data on rice, (the staple food for most Koreans)	Mean UA concentration was significantly higher in males than females (5.8 ± 1.5 vs. 4.4 ± 1.1 mg/dL, *p* < 0.0001). In males and females with hyperuricemia, VC dietary intake was lower than in proper uric acid cases: respectively, 79.7 ± 54.1 vs. 86.0 ± 56.0 mg/day, *p* = 0.01 and 79.2 ± 60.9 vs. 86.0 ± 58.5 mg/day, *p* = 0.02. In total group, the difference was significant too (*p* < 0.001). The observed frequency of hyperuricemia decreased with increased dietary vitamin C intake in male and female subjects after multivariate adjustment (*p* for trend = 0.002 in males and *p* for trend = 0.02 in females). The relationship between total VC intake and the incidence of hyperuricemia was identified in females (*p* for trend = 0.04), but not males (*p* for trend = 0.06).
Ryu KA et al. 2014 South Korea [54]	9010 subjects: 4869 males 4141 females 50.8 ± 9.65 years old	3-days dietary recall (1 weekend and 2 weekdays)	No dietary record or less than 2 days of record Total energy intake <500 kcal or ≥3500 kcal Missing lab test results	Prevalence of hyperuricemia was 13.8% (27.1% men, 5.2% women). Hyperuricemia subjects had significantly lower intakes of VC than controls (57.5 ± 33.5 vs. 69.7 ± 39.8 mg/day, *p* < 0.001).
Zykova SN et al. 2015 Norway Australia [57]	12,765 subjects: 4295 males 5439 females 25–91 years old Data from 2 study cohorts	80-item FFQ/self-designed FFQ	Subjects younger than 25 years old Missing FFQ data or uric acid measurement Known or suspected myocardial infarction or ischemic stroke, pregnancy	In both cohorts mean UA concentration was significantly higher in males than females (5.73 ± 1.27 vs. 4.17 ± 1.0 mg/dL, *p* < 0.0 001, Australian Diabetes, Obesity and Lifestyle Study (AusDiab)) and 6.0 ± 1.42 vs. 4.48 ±1.1 mg/dL, *p* < 0.0001, Tromsø Study). Higher intake of vitamin C was associated with lower serum uric acid (SUA) levels only in females in the Australian cohort; in Norwegian study group, the difference was insignificant. The statistical difference was calculated for the lowest and the highest intake quartiles.
Sun Y et al. 2018 China [55]	14,885 subjects: 7269 males 49.96 ± 17.77 years old 7516 females 49.28 ± 17.18 years old Pooled from three 2-yearcycles	24 h dietary recall collected twice	Subjects younger than 20 years old Missing vitamin C (VC) daily intake data or blood sample Individuals whose total daily energy intake > mean + 3 standard deviations (4630 kcal) or < mean –3 standard deviations	Prevalence of hyperuricemia was 19.1% (21.5% men and 16.8% women). Both total and dietary VC intake was significantly lower in men with hyperuricemia (139 ± 227 vs. 170 ± 302 mg/day, *p* < 0.01 and 82.1 ± 84.9 vs. 93.0 ± 91.7 mg/d, *p* < 0.01) as well as in women subjects (142 ± 230 vs. 160 ± 227 mg/d, *p* = 0.03 and 70.0 ± 60.1 vs. 83.2 ± 72.3 mg/d, *p* < 0.01). Dietary vitamin C and total vitamin C intakes were negatively associated with the risk of hyperuricemia and the increase of OR was linear for successive quartiles.
Zheng Z et al. 2018 US [58]	4576 subjects 1620 males 2956 females 55.5 ± 12.6 years old	158-item FFQ	Missing VC daily intake data or blood sample Taking medication for gout	Prevalence of hyperuricemia was 25.6%. 9% were taking vitamin C supplements. 13% lower of hyperuricemia odds associated with a doubling of vitamin C intake (OR: 0.87, 95% CI: 0.78, 0.97).
So MW et al. 2020 South Korea [56]	10,175 subjects 4200 males 5875 females ≥19 years old, mean 47 years old	single 24 h recall	Clinical history of hemophilia, anticoagulation therapy, chemotherapy in a month, poor vascular access, and age of 80 years or older	There was no statistically significant difference in daily VC intake between hyperuricemia and normouricemia for both men and women participants, respectively, 79 vs. 84 mg/day and 82 vs. 84 mg/day. In analysis of association between hyperuricemia and quartiles of VC intake the OR was significantly decreased with another increase in VC consumption. The highest quartile of dietary VC intake was negatively associated with the risk of hyperuricemia on men: OR = 0.79 (95% CI: 0.63–0.99).

### 3.3. Case-Control Study 

Only one research designed as a case-control study was found in the available literature [62]. The study included a group of 184 Taiwanese citizens, including 92 patients with a history of gout, and an age-matched group of healthy cases. Data on patients’ dietary habits were collected through 24 h recall and 493-item FFQ including the previous year’s data. The questionnaire was focused on microelements and vitamins intake. In the group of healthy people, a significantly higher consumption of fruits and vegetables rich in fiber and vitamin C was shown, which may indicate the potentially protective effect of these ingredients on the risk of developing hyperuricemia and gout. However, it should be noted that although the presented study contained detailed data on eating habits, it did not allow for an accurate assessment of the amount of nutrients such as VC or fiber. In addition, the case-control study design did not allow for a sequence analysis of events, which greatly limited its role in providing strong evidence for a causal relationship between diet and the risk of developing gout. It seems advisable to conduct a similar study on a larger population and compare the obtained results.

### 3.4. Longitudinal and Prospective Study

Due to the extended observation time and the possibility of obtaining uniform information about a study group at different time points, longitudinal studies allow for a reliable determination of the relationship between exposure to environmental factors and the development of a disease. However, this type of examination is not free from disadvantages, such as a decreasing number of observed cases, long time and high costs of the examination, the need to ensure the same standard of diagnostic procedures, and residual confounding. Only three observational studies discussing the relationship between VC dietary consumption and the risk of hyperuricemia or gout were found in the PubMed database (Table 2) [63,64,65]. All studies were conducted in the United States; two of them were based on the same cohort at different time points [63,64]. The Health Professional Follow-up Study (HPFS) was a large and well-characterized prospective cohort designed to study the association between diet and chronic diseases. The total number of study members were 51,529 male dentists, optometrists, osteopaths, pharmacists, podiatrists, and veterinarians.

The follow-up period ranged from one to 20 years, and the total number of observed cases was 50,519 (of which 48,381 were from the HPFS cohort, so it cannot be ruled out that some cases overlapped between the described groups). In the case of studies based on the HPFS cohort [63,64], a standardized 131-item FFQ was used to assess vitamin C intake and an additional set of questions about the intake of dietary supplements, including VC. A study by Beydoun et al. [65] relied on a double, 24 h dietary recall. The determination of SUA levels was made in two smaller studies using enzymatic [63] and spectrophotometric [65] methods, which resulted in differences in the applied definition of hyperuricemia, where, in the case of the study by Gao et al., the upper limit of the norm was 6 mg/dL, while for the study by Beydoun et al., 7 mg/dL for women and 8 mg/dL for men. 

Population-based evidence from the HPFS cohort study [63] showed an association between vitamin C intake and serum uric acid in male subjects, reporting that males with higher VC had lower serum uric acid levels, but it was not a linear correlation—two plateaus were observed: at 90–499 mg/d and then 500 mg/d and higher VC intake. It was observed that higher daily VC intake (containing supplementation) was associated with lower SUA, and the trend was linear (*p* < 0.001), but while patients who supplemented VC were excluded and only dietary intake was taken into consideration, the trend started to be nonsignificant. Exclusion of subjects with gout in clinical history (diagnosis based on self-report) did not change the observed relations; unfortunately, there was a lack of information about anti-gout treatment, such as prolonged allopurinol or febuxostat therapy. 

As in the above-described population, the Beydoun study [65] also showed an inverse correlation between the daily intake of VC and SUA in both men and women. During the second (final) follow-up visit, information on regular intake of dietary supplements was collected; their composition and bioavailability were carefully considered by assessing the actual dose of VC taken. It was shown that regular vitamin C supplementation had a beneficial effect on prolonged SUA levels. The study group included both African Americans and whites and the authors noted that race membership is the most important modifier in these analyses; the results obtained, as in the later cross-sectional study by Zheng et al. [58], were not different for both races. 

The most recent and largest study based on the HPFS cohort and covering twenty years of follow-up of nearly 47,000 respondents was based on a systematic survey conducted every four years since year 1986 [64]. The endpoint of this study was the occurrence of acute gout arthritis, diagnosed according to the American College of Rheumatology survey gout criteria; a detailed interview was collected from respondents with self-reported gout. It is the only field study to assess the direct effect of VC consumption on the risk of developing gout. During the twenty-year follow-up, 1317 new cases of gout were reported. The authors observed a reduction in the risk of developing gout with an increase in the daily intake of VC, both in the diet and associated with pharmacological supplementation. When an intake of up to 250 mg/d was used as the baseline value, then a linear decrease in the risk of developing the disease was observed: RR for a dose of 500–999 mg/d was 0.83 (95% CI: 0.71 to 0.97); RR for a dose of 1000–1499 mg/d was 0.66 (95% CI: 0.52 to 0.86), and RR for a dose ≥1500 mg/day was 0.55 (95% CI: 0.38 to 0.80) (*p* for trend < 0.001). It should be noted that the average daily dose of vitamin C 250 mg/d corresponds with the upper quartile of ascorbic acid intake observed in other studies [55,56,57], while here it is the lowest daily dose.

The studies described above are difficult to compare due to methodological differences and the fact that two of them were based on the same initial population. However, although they repeatedly indicate the beneficial effect of a higher daily intake of vitamin C on the risk of hyperuricemia and the development of gout, it is difficult to indicate the minimum dose of daily VC intake. It seems that 500 mg/d is a safe dose; moreover, as observed by Gao et al. [63], a plateau effect between a dose of 499–1000 mg raises doubts about the sense of daily dose escalating. On the other hand, in the study by Choi et al. [64], the benefit from a dose even as high as >1500 mg/d was statistically significant. Further observational and iterative studies in a more diverse population are required to determine the best dose and ultimately prove the efficacy of VC in reducing the risk of hyperuricemia. The available studies, although promising, cannot be treated as conclusive evidence of the relationship between the VC intake and SUA level.

### 3.5. Interventional Studies

The last type of research in humans to be discussed will be international studies, which are the only ones that can determine the direct clinical benefit of using a drug or dietary supplement in the treatment or prevention of disease development, in this case, hyperuricemia and gout. Fifteen clinical research trials were identified in the available literature (Table 3). The oldest [44,46,47] were focused on the uricosuric effect of vitamin C supplementation. Further work focused on SUA levels in the healthy population and patients with various chronic diseases, including end-stage renal disease [66,67,68,69,70,71,72,73,74]. The last subgroup consisted of studies where patients with gout in their clinical history were included [44,75,76]. All studies were relatively short and lasted from a few hours to a maximum of three months. Supplemented doses of VC ranged from 500 mg/day to as much as 12 g. Despite the use of even high doses of VC, only one patient was excluded from a study due to poor drug tolerance. Conducting clinical trials with VC—even at high doses—appears to be safe due to relatively mild overdose symptoms, such as osmotic diarrhea and nausea, since the excess VC is excreted with urine. Due to the long time that elapsed since the publication of the first of the discussed studies, the methodology of the laboratory assessment of SUA and UA excreted in urine differs significantly between studies, which makes it difficult to explore them directly.

The first subgroup of the presented studies was carried out in the 1970s and 1980s and assessed the change in the concentration of uric acid excreted due to the supplementation of ascorbic acid [44,46,47]. The studies were characterized by a very small number of patients enrolled, while the control was based on the results obtained before the intervention. For the first study of this type by Stein et al. [44], 14 people were included, including five patients with gout, three with hyperuricemia, and six healthy people; the age of the respondents and the information on the sex distribution in each group are unknown. The lack of information on the sex distribution may make the interpretation of the obtained data difficult, because in later observations, differences in uric acid metabolism between women and men were shown, which could be due to differences in the level of sex-related hormones [60]. During the study, patients received different doses of VC (from 500 mg to 4 g) in monotherapy or in combination with aspirin or pyrazinamide, separated by 48 h intervals; three patients were also subjected to several days (3–7 days) of observation, during which they took 8 g VC/d [44]. The authors observed a significant direct effect of high dose of VC on the increase in the ratio of uric acid clearance to creatinine clearance in the kidney and also described the relationship between the increase in filtration (assessed 3 h after VC intake) and the dose taken—see Table 3. In extended follow-up, despite the high dose of 8g/d VC, the increase in UA filtration was slightly lower. 

Another analyzed subgroup of studies are experiments aimed at assessing the effect of ascorbic acid supplementation on the level of uric acid in the blood serum in healthy people. In most of the presented studies, VC was supplemented at a dose of 500 mg/d [66,68,74], one VC was supplemented at a dose of 1000 mg/d [67]; all studies were double-blind, placebo-controlled studies. The first study by Baser et al. [66] showed no significant reduction in SUA. The results obtained in the remaining described groups are in contrast with this study. After two-month supplementation of ascorbic acid at a dose of 500 mg/d in 92 healthy volunteers, a statistically significant decrease in SUA levels was observed [68], and the decrease in UA concentration was inversely proportional to the increase in serum ascorbic acid concentration. At the same time, a nonsignificant increase in SUA was observed in the placebo-treated group. A stronger effect of VC was observed in the subgroup of 21 patients with hyperuricemia at baseline; here, the decrease in SUA was 1.5 mg/dL [68]. A stronger reduction of SUA levels in patients with a higher concentration of UA at the beginning was also confirmed in other reports [69,73]. 

Only young men who, apart from VC supplementation with 1000 mg [67] or 500 mg/d [74], were subjected to intensive physical training, and being at high altitude [74], were qualified for the remaining two studies. In both cases, the effect of VC supplementation was to reduce the increase in SUA associated with accelerated metabolism resulting from increased physical exertion. Elevated plasma urate levels are commonly observed in physically active subjects [77]. In the second identically designed study presented by the team of Pang et al. [74], supplementation with 75 IU/d of vitamin E was used in the control instead of placebo. The strong antioxidant effect prevented hyperuricemia caused by high altitude and exercise. Then, after a single intake of 4 g VC (202 ± 41 vs. 174 ± 24% of baseline clearance), the hyperuricosuria effect persisted 1–2 days after discontinuation of VC supplementation. In 2 patients who were followed up for several days in the daily measurements of SUA, no linear decrease was observed; additionally, no stable increase in renal UA clearance was observed. However, at the end of the observation, the SUA level decreased by 1.2–3.1 mg/day. There was no separate analysis of healthy and gout patients in the description of the study [44]. A similar outcome of increased uricosuria was observed in the study of Sutton et al. [47]. Unfortunately, we were not able to obtain all the full texts of the cited publications, and in the case of the research by Sutton et al. and Baser, the data were collected only on the basis of available abstracts, which makes it impossible to analyze the above-mentioned studies in detail. In contrast to the experiences of the Stein [44] and Sutton [47] syndromes, in the results obtained in the study by Mitch et al. [46], six healthy volunteers took part in the experiment and took a dose of 4 or 12 g of VC in four divided doses throughout the day for a month. In this group, there were no significant changes in the level of SUA or in the increased uricosuric effect. The relatively short observation period of the above-described studies limits their role as evidence for preventive properties of VC. However, some of them suggest a beneficial role of vitamin C supplementation in reducing SUA levels amongst healthy adults and, consequently, hyperuricemia. Further research is required in this field.

Subjects with additional clinical diseases were qualified for further five studies [69,70,71,72,73]. These studies differed significantly in terms of the protocol used; however, in all of them, a positive effect of VC supplementation was achieved in variable doses from 250 mg administered intravenously three times a week to 1 g/d taken orally to reduce the concentration of UA in the blood serum. A study by El Mashad et al. [72] presented sixty children with end-stage renal disease who were dialyzed three times a week. For three months, thirty children received 250 mg of VC intravenously, while the remaining thirty received saline solution. In the study group, there was a significant decrease in the level of uric acid (8.33 ± 1.61 vs. 5.95 ± 0.75, *p* < 0.0001) compared to the baseline value. A similar result was obtained in an analogical study conducted in a population of dialyzed adults [71], where after two months of systematic supplementation with 250 mg VC intravenously three times a week after dialysis, the mean SUA value decreased from 6.2 mg/dL to 5.8 mg/dL (*p* = 0.02). In patients from the control group, the SUA level remained unchanged. 

The last group discussed in this review is the clinically most interesting study focused on patients with gout [44,75,76]. These patients often require chronic pharmacotherapy and should follow a low-purine diet for a long time and avoid alcohol and smoking. The issue of additional reduction of the risk of recurrent arthritis seems to be particularly important in this group. In the literature, there are three intervention studies in patients with gout. The most important one, established in 1976, is the experiment by Stein et al. [44], in which the health effect of vitamin C was assessed. Five people with gout qualified for this study, but there are no detailed results for this group; the study is described above. Two other studies did not show a significant reduction in uric acid concentration in patients with gout [75,76] who were supplemented with vitamin C at a dose of 500 mg/day for two months but for whom stabilization of the SUA level was demonstrated. A study by Stamp et al. compared the effect of supplementation with vitamin C with the addition of allopurinol therapy—modifying its current dose or adding VC to the current dose of allopurinol. A significant decrease in SUA was demonstrated in all groups using allopurinol, while in the group that used only VC supplementation, uric acid concentration was stabilized; there were no significant differences in the clinical efficacy of allopurinol as monotherapy or in combination with VC. It should be noted that a dose of 500 mg VC determined at the beginning of the study remained unchanged throughout the two months of observation, while the dose of allopurinol was actively changed depending on the control of uric acid levels. In the group of 10 people who were included in the allopurinol therapy for the first time, the initial dose was on average 95 mg/d at the beginning, and it reached 210 mg/d at the end of the study, while in those who received therapy earlier, the initial dose was on average 345 mg/d and was 385 mg/d at the end of the study. However, the observational study by Choi et al. [64] showed a beneficial effect of an increased dose of VC even above 1.5 g/d. Perhaps, when using VC as an adjunct to urate-lowering therapy, the use of higher doses of ascorbic acid should be considered in order to obtain its stronger therapeutic effect.

The summarized results of the above studies can be found in the meta-analysis prepared by Juraszek et al. [78]. The team analyzed data obtained from 13 randomized clinical trials, with a total number of 556 participants receiving a vitamin C in a median dose of 500 mg/d by a mean of 30 days. They concluded that supplementation of VC reduced serum concentration of uric acid −0.35 mg/dL (*p* = 0.032). 

Currently, there is no evidence for the usefulness of vitamin C supplementation in patients with gout, but its usefulness in lowering uric acid levels in patients who have not yet developed arthritis may be considered. It seems advisable to conduct further clinical trials with a longer duration of vitamin supplementation and with various doses in order to establish the optimal dose. Perhaps the recommendation of additional vitamin C supplementation in people with hyperuricemia will find its application in everyday clinical practice.

## 4. Conclusions

The effect of diet on serum uric acid levels and the development of acute arthritis in a person with gout is well documented. However, it seems that the problem is not only related to the consumption of meat products and alcohol but is extremely complex and multifactorial. It is difficult to clearly identify all nutrients that play a role in the etiopathogenesis of the disease. Although the literature contains the results of numerous studies reporting on the relationship between serum concentration of vitamin C and uric acid, available data do not allow for an unambiguous assessment of the usefulness of ascorbic acid supplementation in the prevention and treatment of gout. It seems that a higher level of vitamin C in the serum has a positive effect on purine metabolism and favors the reduction of uric acid level, thus reducing the risk of monosodium urate crystal deposition in joints structures and soft tissue. However, there is too little evidence of a beneficial effect of ascorbic acid supplementation in the prevention and treatment of gout, as well as for its usefulness during an exacerbation of the disease. Some research suggests its potential preventive role but studies are limited due to their short observation period. Long-term follow-up of a wide patient population and prolonged intervention studies are necessary to draw final conclusions. Currently, it seems that the most clinically sound rationale is to recommend a balanced diet rich in vegetables, fruits, and cereals, with a limited content of meat and meat products. In addition, it is necessary to maintain a proper body mass and physical activity and to stop smoking.

## Figures and Tables

**Table 2:** Longitudinal studies discussing the dependence of vitamin C intake in the diet and the risk of hyperuricemia.

Study	Study Population	Follow-Up Duration	Dietary Assessment	Exclusion Criteria and End Point	Results
Gao X et al. 2008 US [63]	1387 men Age 40–75 years old	FFQ was collected every two years for 8 years before blood sample collection 8 years	131-item FFQ	BMI ≥ 30kg/m^2^, hypertension, blood sample drawn after fasting < 8 h at the beginning of the study	Greater VC supplement intake was significantly associated with a lower serum uric acid (*p* for trend < 0.001). Although higher dietary VC intake categories tended to have lower serum uric acid levels than the lowest category, the linear trend was not significant (*p* for trend = 0.10). The multivariate ORs for hyperuricemia across total VC intake categories were 1 (reference), 0.58, 0.57, 0.38, and 0.34 (95% CI: 0.20–0.58; *p* for trend < 0.001). The multivariate OR for the highest versus lowest categories of total VC intake was 0.31 (95% CI: 0.17–0.56, *p* for trend = 0.009).
Choi HK et al. 2009 US [64]	46,994 men Age 40–75 years old	20 years vitamin C intake was verified every 4 years	>130-item FFQ	Gout in history End point–case of gout	During the 20 years of follow-up, 1317 cases of gout incidents were confirmed Relative risk of gout in cases of VC intake < 250mg/day was 0.83 (95% confidence interval CI: 0.71 to 0.97) for total VC intake 500–999 mg/day; 0.66 (0.52 to 0.86) for 1000–1499 mg/day, and 0.55 (0.38 to 0.80) for ≥1500 mg/day (*p* for trend < 0.001).
Beydoun MA et al. 2018 US [65]	2138 participants 1238 African American, 900 white urban adults 973 males 1165 females aged 30–64 years old 1208 women 930 men	1–8 years Mean 4.64 ± 0.93 years	24 h dietary recall collected twice and dietary supplements questionnaire adapted from NHANES 2007–08	x	Supplemental vitamin C may have putative protective effects among both whites and African Americans.

NHANES: The National Health and Nutrition Examination Survey.

**Table 3:** Interventional studies discussing the dependence of vitamin C supplementation and the risk of hyperuricemia, gout, and uricosuric effect.

Study	Study Population	Follow-Up Duration	Intervention	Exclusion Criteria	Results
Stein HB et al. 1976 Canada [44]	14 subjects 10 males and 4 females 5 with gout, 3 hyperuricemia, 6 controls	8 h/ 3–7 days	Diet: 2600 kcal, 70 g of protein, purine free VC 0.5, 2.0 and 4.0 g VC 4.0 g + ASA 600mg CV 4.0 g ASA 600 mg VC 4.0 g + pyrazinamide 3.0 g Pyrazinamide 3.0 g 48 h brake between experiments 3 subjects administrated 8.0 g of VC by 3–7 days	Drug known to interfere with uric acid metabolism or excretion.	4.0 g VC intake caused maximum increase to 202 ± 41% uric acid clearance. The peak effect varied between 6 to 8 h (*p* < 0.01). 2.0 g VC intake increase to 152 ± 24% UA clearance (*p* > 0.05). 0.5 g VC intake increase to 128 ± 6% UA clearance (*p* > 0.05). ASA intake decreased UA clearance. In prolonged VC administration, the UA clearance was increased to 174% ± 24% (*p* < 0.01) of the control values, and the effect was maintained for 1 to 2 days thereafter. In this group the serum uric acid decreased by 1.2 to 3.1 mg/dL because of a sustained uricosuria.
Mitch WE et al. 1981 US [46]	6 healthy subjects 4 males 2 females Age 22–42 years old	1month	4 or 12 g of VC in 4 separate doses/ day	History of gout, hypertension, renal disease, or other diseases associated with abnormal uric acid metabolism No drugs or vitamins were taken for at least 1 week before study	There was no effect on serum uric acid concentration or uric acid excretion and clearance by the kidney.
Sutton JL et al. 1983 * UK [47]	16 healthy subjects	7 days	1 g of VC 3 times/day	Data not available	Transient increase in uric acid excretion was observed.
Baser E* 1991 Turkey [66]	152 healthy subjects 20.0 ± 0.38 years old	1 month	105 subjects: 500 mg of VC once/day 47 subjects: placebo	Data not available	There was no significant effect of VC on plasma uric acid concentration (*p* > 0.05).
Yanai H et al. 2004 Japan [67]	8 well-trained male athletes	3 weeks	4 subjects: 1 g VC 4 subjects: placebo unified diet and training program		After training in the placebo group, significant increase of UA serum concentration was observed (4.48 ± 0.83 mg/dL before training vs. 6.15 ± 0.47 mg/dL in the end of the study, *p* < 0.05). In VC supplementing group, the UA concentration stayed stable (5.98 ± 0.67 vs. 5.13 ± 0.52 mg/dL; *p* > 0.05).
Huang HY et al. 2005 US [68]	184 subjects 74 males 110 females Age 58.15 ± 13.65 years old	2 months	92 subjects: 500 mg of VC once/day 92 subjects: placebo	Regular exposure to passive tobacco smoke for ≥1 h/ day or consumption of ≥14 servings of alcoholic beverages/week. 2-month period of supplement abstinence before study beginning was required.	Baseline VC dietary intake was equal. At the end of the supplementation period, the serum UA concentration was significantly reduced in the active vitamin C group but not in the placebo group (mean change 0.09, 95% CI: −0.05 to 0.2 vs. −0.5, 95% CI: −0.6 to −0.3, *p*< 0.0001). Serum UA was inversely correlated with changes in serum ascorbic acid −0.32 (*p* < 0.0002) in the VC group. Among persons who were hyperuricemic at baseline (*n* = 21), VC supplementation reduced serum UA by a mean of 1.5 mg/dL (*p =* 0.0008).
Naziroğlu M et al. 2009 Turkey [49]	120 subjects 40 non-diabetic postmenopausal women 40 postmenopausal women with DM2 Age 45–65 years old 40 young controls Age 19–28 years old	1.5 months	40 control subjects–without intervention 40 postmenopausal non-diabetic subjects–HRT 20 postmenopausal diabetic subjects-HRT 20 postmenopausal diabetic subjects–HRT + 1 g VC and 600 mg VE	Women taking insulin or lipid lowering therapy or antioxidant vitamins within the last 6 months or HRT within the last 3 months Control group had not been taking oral contraceptives for at least 6 months before the blood sample collection	The UA concentration in group of postmenopausal diabetic women was significantly higher than in control group (3.4 ± 0.1 vs. 2.4 ± 0.5 mg/dL, *p* < 0.01). In this group after 6 weeks of HRT together with vitamins C and E supplementation, a significant decrease of UA serum concentration compared to baseline was observed (3.4 ± 0.1 vs. 2.9 ± 0.8 mg/dL, *p* < 0.05).
Hunter DC et al. 2011 New Zeland [70]	48 smokers 18 males 25 females Age 44.3 ± 8.1 years old	1.5 months	Nonsupplemented milk Prototype supplemented milk (i.e., 200mg VC/serve) 200mL of milk twice a day	Younger than 30 years old Chronic disease (e.g., diabetes, cardiovascular disease) Taking medications known to interfere with the immune system used nutritional supplements	In supplemented milk group, serum UA concentration was significantly lower after 6 weeks than in baseline (*p* = 0.026), and fasting concentrations of plasma vitamin C were significantly higher (*p* < 0.001).
Stamp LK et al. 2013 New Zeland [75]	40 patients with gout or hyperuricemia 36 males 4 females Mean age 58.1 years old	2 months	10 subjects: Start allopurinol 50–100 mg/d (after 8 weeks the dose range 100–300 mg/day) 10 subjects: VC 500 mg/d 10 subjects: increase previous allopurinol dose (final dosage 150–500 mg/day) 10 subjects: allopurinol in dose as before + VC 500 mg/d	Taking over-the-counter vitamin supplements	There was no significant reduction of UA concentration between baseline and final sampling in group of patients with supplementation of VC. The reduction in UA levels was significantly lower in the 20 patients receiving VC compared to those who started receiving or increased the dose of allopurinol (mean reduction 0.23 vs. 1.98 mg/dL; *p <* 0.001). Results showed that supplemental VC at a dosage of 500 mg/day did not lead to a clinically significant reduction in the UA level in patients with gout.
Binaz V et al. 2014 Iran [71]	172 hemodialysis patients 102 males 63 females Age 61.54 ± 12.72 years old	2 months	59 subjects: 250 mg of VC 3 times/week 58 subjects: Placebo 55 subjects: No intervention	7 were excluded from the study due to transition to other dialysis centers, being infected by active infections, cancer, death, or refusal to continue participation	46.7% patients had hyperuricemia. At baseline, the UA concentration was similar in all study groups. After 8 weeks of study, UA serum concentration was significantly lower in VC supplemented group than in both controls (5.8 ± 1.3 vs. 6.4 ± 1.3 and 6.3 ± 1.1 mg/dL, *p* = 0.02). In patients with hyperuricemia (*n* = 80), the decrease of UA after 8 weeks of VC supplementation was significantly lower than in both controls (5.90 ± 1.30 vs. 6.80 ± 0.64 and 6.80 ± 1.01 mg/dL, *p* = 0.004).
El Mashad GM et al. 2016 [72]	60 children with end-stage renal disease 29 males 31 females Age 8.85 ± 10.2	3 months	30 subjects: 250 mg VC i.v. 3 times/week 30 subjects: placebo (saline i.v.)	Primary (nonuremic) cardiovascular disease Taking VC supplementation during the last three months Participants in another clinical trial	Serum mean uric acid levels were 8.06 ± 1.8 mg/dL and ascorbic acid levels were 8.90 ± 4.06 μmol/L (norm in children > 28.4 μmol/L). In the supplemented group, statistically significant increase of VC serum concentration was observed (8.97 ± 4.38 vs. 22.06 ± 9.59 μmol/L, *p* < 0.0001) as well as significant decrease of UA concentration (8.33 ± 1.61 vs. 5.95 ± 0.75 mg/dL, *p* < 0.0001) between baseline and final sampling.
Choudhury MR et al. 2016 Bangladesh [73]	71 subjects–patients with musculoskeletal disorder except gout 25 males 46 females Age 34.13 ± 13.5 years old	3 months	37 subjects: VC 500 mg/d 34 subjects: placebo	Gout or any malignant disorder in clinical history Smokers and heavy drinkers	In VC supplementing group, mean decrease of serum UA was −0.32 mg/dL (95% CI: −0.73 to 0.77), while in the placebo group, the mean change was + 0.12 mg/dL (95% CI: −0.22 to 0.47). Subjects were split into subgroups uniform in terms of sex, BMI, and baseline UA concentration—patients with a higher serum UA level had greater benefit from VC supplementation.
Azzech FS et al. 2017 Saudi Arabia [76]	30 subjects 15 with gout 52.85 ± 11.36 years old 15 with hyperuricemia 54.23 ± 12.26 years old 16 males 14 females Aged 24–75 years old	2 months	All subjects: VC 500 mg/d	Patients less than 20 years, history of dialysis, alcohol consumption, pregnant or lactating women, multivitamin supplements during the last three months, and diuretic drug and/or any uricosuric agent	After 8 weeks in gout group, the mean UA concentration was nonsignificantly higher than at the baseline (8.4 ± 1.15 vs. 8.09 ± 1.09 mg/dL, *p* > 0.05), while in hyperuricemia group, a significant decrease was observed (7.16 ± 1.04 vs. 7.94 ± 0.93 mg/dL, *p* < 0.05). The reduction of UA was slightly higher for women than men in hyperuricemia group.
Peng H et al. 2018 China [74]	66 male army recruits Age 19.25 ± 1.5 years old	1 month	33 subjects: VC 500 mg/d 33 subjects: placebo	Any chronic disease and long-term medication necessity Previously lived at an altitude > 2500 m Not completing follow-up	At 1 month, UA concentration was significantly higher than at baseline (7.33 ± 1.33 vs. 6.02 ± 1.34 mg/dL, *p* < 0.001); prevalence of hyperuricemia was also significantly higher (63.6 vs. 19.7%, *p* < 0.001). In VC supplemented group, both the level of serum UA (6.92 ± 1.25 vs. 7.75 ± 0.92 mg/dL, *p* = 0.003) and the prevalence of hyperuricemia (48.5 vs. 78.8%; *p* = 0.020) were lower.
Peng H et al. 2018 China [74]	120 male army recruits	1 month	57 subjects: VC 500 mg/d 58 subjects: VE 75 IU/d	Any chronic disease and long-term medication necessity Previously lived at an altitude > 2500 m Not completing follow-up	At 1 month, UA concentration was significantly higher than at baseline (7.32 ± 1.37 vs. 6.85 ± 1.19 mg/dL, *p* = 0.053), and a higher prevalence of hyperuricemia (59.6 vs. 43.1%, *p* = 0.076) was observed. Both were higher in the vitamin C group relative to the vitamin E group, but the differences were not statistically significant.

## Data Availability

Data available in a publicly accessible repository.

## References

[1] Padayatty S.J., Levine M. (2016). Vitamin C: The known and the unknown and Goldilocks. Oral Dis..

[2] Grzybowski A., Pietrzak K. (2013). Albert Szent-Györgyi (1893–1986): The scientist who discovered vitamin C. Clin. Dermatol..

[3] Lykkesfeldt J., Tveden-Nyborg P. (2019). The Pharmacokinetics of Vitamin C. Nutrients.

[4] Carpenter K.J. (2012). The Discovery of Vitamin C. Ann. Nutr. Metab..

[5] Maxfield L., Crane J.S. (2020). Vitamin C Deficiency.

[6] Granger M., Eck P. (2018). Dietary Vitamin C in Human Health. Adv. Food Nutr. Res..

[7] Sauberlich H.E. (1994). Pharmacology of Vitamin C. Annu. Rev. Nutr..

[8] Chambial S., Dwivedi S., Shukla K.K., John P.J., Sharma P. (2013). Vitamin C in Disease Prevention and Cure: An Overview. Indian J. Clin. Biochem..

[9] Padayatty S.J., Katz A., Wang Y., Eck P., Kwon O., Lee J.-H., Chen S., Corpe C., Dutta A., Dutta S.K. (2003). Vitamin C as an Antioxidant: Evaluation of Its Role in Disease Prevention. J. Am. Coll. Nutr..

[10] Frei B., England L., Ames B.N. (1989). Ascorbate is an outstanding antioxidant in human blood plasma. Proc. Natl. Acad. Sci. USA.

[11] Namas R., Meysami A., Siegal D., Rubin B. (2014). Gout and ultrasound: The disease of kings and the queen of imaging. Gout Hyperuricemia.

[12] Hansildaar R., Vedder D., Baniaamam M., Tausche A.-K., Gerritsen M., Nurmohamed M.T. (2021). Cardiovascular risk in inflammatory arthritis: Rheumatoid arthritis and gout. Lancet Rheumatol..

[13] Rahimi-Sakak F., Maroofi M., Rahmani J., Bellissimo N., Hekmatdoost A. (2019). Serum uric acid and risk of cardiovascular mortality: A systematic review and dose-response meta-analysis of cohort studies of over a million participants. BMC Cardiovasc. Disord..

[14] Li L., Zhang Y., Zeng C. (2020). Update on the epidemiology, genetics, and therapeutic options of hyperuricemia. Am. J. Transl. Res..

[15] Roman Y.M., Daniel K. (2019). Inouye College of Pharmacy Scripts: Perspectives on the Epidemiology of Gout and Hyperuricemia. Hawaii J. Med. Public Health..

[16] Chen L., Han S., Liu F., Chen S., Chen X., Chen H. (2020). Global prevalence of hyperuricemia in adolescents from 2000 to 2019: A meta-analysis. BMC Pediatrics.

[17] Safiri S., Kolahi A., Cross M., Hill C., Smith E., Carson-Chahhoud K., Mansournia M.A., Almasi-Hashiani A., Ashrafi-Asgarabad A., Kaufman J. (2020). Prevalence, deaths and disability adjusted life years (DALYs) due to musculoskeletal disorders for 195 countries and territories 1990-2017. Arthritis Rheumatol..

[18] Chowalloor P.V., Keen H.I. (2013). A systematic review of ultrasonography in gout and asymptomatic hyperuricaemia. Ann. Rheum. Dis..

[19] Revaz S., Dudler J. (2007). Clinical manifestations of gout. Rev. Med. Suisse.

[20] Roddy E., Doherty M. (2010). Gout. Epidemiology of gout. Arthritis Res..

[21] Johnson R.J., Titte S., Cade J.R., Rideout B.A., Oliver W.J. (2005). Uric acid, evolution and primitive cultures. Semin. Nephrol..

[22] Wu X.W., Lee C.C., Muzny D.M., Caskey C.T. (1989). Urate oxidase: Primary structure and evolutionary implications. Proc. Natl. Acad. Sci. USA.

[23] Wu X., Muzny D.M., Lee C.C., Caskey C.T. (1992). Two independent mutational events in the loss of urate oxidase during hominoid evolution. J. Mol. Evol..

[24] Oda M., Satta Y., Takenaka O., Takahata N. (2002). Loss of Urate Oxidase Activity in Hominoids and its Evolutionary Implications. Mol. Biol. Evol..

[25] Kratzer J.T., Lanaspa M.A., Murphy M.N., Cicerchi C., Graves C.L., Tipton P.A., Ortlund E.A., Johnson R.J., Gaucher E. (2014). Evolutionary history and metabolic insights of ancient mammalian uricases. Proc. Natl. Acad. Sci. USA.

[26] Johnson R.J., Sautin Y.Y., Oliver W.J., Roncal C., Mu W., Sanchez-Lozada L.G., Rodriguez-Iturbe B., Nakagawa T., Benner S.A. (2008). Lessons from comparative physiology: Could uric acid represent a physiologic alarm signal gone awry in western society?. J. Comp. Physiol. B.

[27] Ames B.N., Cathcart R., Schwiers E., Hochstein P. (1981). Uric acid provides an antioxidant defense in humans against oxidant- and radical-caused aging and cancer: A hypothesis. Proc. Natl. Acad. Sci. USA.

[28] Shen L., Ji H.-F. (2011). Potential of vitamin C in the prevention and treatment of gout. Nat. Rev. Rheumatol..

[29] Shulten P., Thomas J., Miller M., Smith M., Ahern M. (2009). The role of diet in the management of gout: A comparison of knowledge and attitudes to current evidence. J. Hum. Nutr. Diet..

[30] Choi H.K., Liu S., Curhan G. (2005). Intake of purine-rich foods, protein, and dairy products and relationship to serum levels of uric acid: The Third National Health and Nutrition Examination Survey. Arthritis Rheum..

[31] Kobylecki C.J., Afzal S., Nordestgaard B.G. (2018). Genetically high plasma vitamin C and urate: A Mendelian randomization study in 106 147 individuals from the general population. Rheumatol..

[32] Kim S.-K., Choe J.-Y., Park K.-Y. (2019). TXNIP-mediated nuclear factor-κB signaling pathway and intracellular shifting of TXNIP in uric acid-induced NLRP3 inflammasome. Biochem. Biophys. Res. Commun..

[33] Choe J.-Y., Kim S.-K. (2017). Quercetin and Ascorbic Acid Suppress Fructose-Induced NLRP3 Inflammasome Activation by Blocking Intracellular Shuttling of TXNIP in Human Macrophage Cell Lines. Inflammation.

[34] Linster C.L., Van Schaftingen E. (2007). Vitamin C: Biosynthesis, recycling and degradation in mammals. FEBS J..

[35] Yang H. (2013). Conserved or Lost: Molecular Evolution of the Key Gene GULO in Vertebrate Vitamin C Biosynthesis. Biochem. Genet..

[36] Shah A., Keenan R.T. (2010). Gout, Hyperuricemia, and the Risk of Cardiovascular Disease: Cause and Effect?. Curr. Rheumatol. Rep..

[37] Duplancic D., Kukoc-Modun L., Modun D., Radic N. (2011). Simple and Rapid Method for the Determination of Uric Acid-Independent Antioxidant Capacity. Molecules.

[38] Kirschbaum B. (2001). Renal regulation of plasma total antioxidant capacity. Med. Hypotheses.

[39] Fukae J., Fujioka S., Yanamoto S., Mori A., Nomi T., Hatano T., Fukuhara K., Ouma S., Hattori N., Tsuboi Y. (2017). Serum uric acid level is linked to the disease progression rate in male patients with multiple system atrophy. Clin. Neurol. Neurosurg..

[40] Kim W.-J., Kim H.R., Song J.S., Choi S.T. (2020). Low levels of serum urate are associated with a higher prevalence of depression in older adults: A nationwide cross-sectional study in Korea. Arthritis Res..

[41] Keizman D., Ish-Shalom M., Berliner S., Maimon N., Vered Y., Artamonov I., Tsehori J., Nefussy B., Drory V.E. (2009). Low uric acid levels in serum of patients with ALS: Further evidence for oxidative stress?. J. Neurol. Sci..

[42] Luo X.L.J.J. (2013). A Double-edged Sword: Uric Acid and Neurological Disorders. Brain Disord. Ther..

[43] Beydoun M.A., Canas J.-A., Fanelli-Kuczmarski M.T., Tajuddin S.M., Evans M.K., Zonderman A.B. (2017). Genetic risk scores, sex and dietary factors interact to alter serum uric acid trajectory among African-American urban adults. Br. J. Nutr..

[44] Stein H.B., Hasan A., Fox I.H. (1976). Ascorbic Acid-Induced Uricosuria. A Consequency of Megavitamin Therapy. Ann. Intern. Med..

[45] Berger L., Gerson C.D., Yü T.-F. (1977). The effect of ascorbic acid on uric acid excretion with a commentary on the renal handling of ascorbic acid. Am. J. Med..

[46] Mitch W.E., Johnson M.W., Kirshenbaum J.M., Lopez R.E. (1981). Effect of large oral doses of ascorbic acid on uric acid excretion by normal subjects. Clin. Pharmacol. Ther..

[47] Sutton J.L., Basu T.K., Dickerson J.W. (1983). Effect of large doses of ascorbic acid in man on some nitrogenous components of urine. Hum. Nutr. Appl. Nutr..

[48] Enomoto A., Kimura H., Chairoungdua A., Shigeta Y., Jutabha P., Cha S.H., Hosoyamada M., Takeda M., Sekine T., Igarashi T. (2002). Molecular identification of a renal urate–anion exchanger that regulates blood urate levels. Nat. Cell Biol..

[49] Torralba K.D., De Jesus E., Rachabattula S. (2012). The interplay between diet, urate transporters and the risk for gout and hyperuricemia: Current and future directions. Int. J. Rheum. Dis..

[50] Choi H.K., Mount D.B., Reginato A.M. (2005). Pathogenesis of Gout. Ann. Intern. Med..

[51] Thangaraju M., Ananth S., Martin P.M., Roon P., Smith S.B., Sterneck E., Prasad P.D., Ganapathy V. (2006). c/ebpδ Null Mouse as a Model for the Double Knock-out of slc5a8 and slc5a12 in Kidney. J. Biol. Chem..

[52] Nakagawa T., A Lanaspa M., Johnson R.J. (2019). The effects of fruit consumption in patients with hyperuricaemia or gout. Rheumatol..

[53] Bae J., Shin D.H., Chun B.-Y., Choi B.Y., Kim M.K., Shin M.-H., Lee Y.-H., Park P.S., Kim S.-K. (2014). The effect of vitamin C intake on the risk of hyperuricemia and serum uric acid level in Korean Multi-Rural Communities Cohort. Jt. Bone Spine.

[54] Ryu K.A., Kang H.H., Kim S.Y., Yoo M.K., Kim J.S., Lee C.H., Wie G.A. (2014). Comparison of Nutrient Intake and Diet Quality Between Hyperuricemia Subjects and Controls in Korea. Clin. Nutr. Res..

[55] Sun Y., Sun J., Wang J., Gao T., Zhang H., Ma A. (2018). Association between vitamin C intake and risk of hyperuricemia in US adults. Asia Pac. J. Clin. Nutr..

[56] So M.W., Lim D.H., Kim S.H., Lee S. (2020). Dietary and nutritional factors associated with hyperuricemia: The seventh Korean National Health and Nutrition Examination Survey. Asia Pac. J. Clin. Nutr..

[57] Zykova S.N., Storhaug H.M., Toft I., Chadban S.J., Jenssen T.G., White S.L. (2015). Cross-sectional analysis of nutrition and serum uric acid in two Caucasian cohorts: The AusDiab Study and the Tromsø study. Nutr. J..

[58] Zheng Z., Harman J.L., Coresh J., Köttgen A., McAdams-DeMarco M.A., Correa A., Young B.A., Katz R., Rebholz C.M. (2018). The Dietary Fructose:Vitamin C Intake Ratio Is Associated with Hyperuricemia in African-American Adults. J. Nutr..

[59] Beck K.L., Houston Z.L., McNaughton S.A., Kruger R. (2018). Development and evaluation of a food frequency questionnaire to assess nutrient intakes of adult women in New Zealand. Nutr. Diet..

[60] Poletto J., Harima H.A., Ferreira S.R.G., Gimeno S.G.A. (2011). Hyperuricemia and associated factors: A cross-sectional study of Japanese-Brazilians. Cadernos de Saúde Pública.

[61] Choi H.K., Willett W., Curhan G. (2010). Fructose-Rich Beverages and Risk of Gout in Women. JAMA.

[62] Lyu L.-C., Hsu C.-Y., Yeh C.-Y., Lee M.-S., Huang S.-H., Chen C.-L. (2003). A case-control study of the association of diet and obesity with gout in Taiwan. Am. J. Clin. Nutr..

[63] Gao X., Curhan G., Forman J.P., Ascherio A., Choi H.K. (2008). Vitamin C intake and serum uric acid concentration in men. J. Rheumatol..

[64] Choi H.K., Gao X., Curhan G. (2009). Vitamin C Intake and the Risk of Gout in Men. Arch. Intern. Med..

[65] Beydoun M.A., Fanelli-Kuczmarski M.T., Canas J.-A., Beydoun H.A., Evans M.K., Zonderman A.B. (2018). Dietary factors are associated with serum uric acid trajectory differentially by race among urban adults. Br. J. Nutr..

[66] Beser E. (1991). The effects of short-term vitamin C on plasma bun, uric acid, cholesterol and triglyceride levels. Acta medica Hung..

[67] Yanai H., Morimoto M. (2004). Effect of ascorbate on serum lipids and urate metabolism during exhaustive training. Clin. Sci..

[68] Huang H.-Y., Appel L.J., Choi M.J., Gelber A.C., Charleston J., Norkus E.P., Miller E.R. (2005). The effects of vitamin C supplementation on serum concentrations of uric acid: Results of a randomized controlled trial. Arthritis Rheum..

[69] Nazıroğlu M., Simsek M., Naziroğlu M. (2009). Effects of hormone replacement therapy with vitamin C and E supplementation on plasma thyroid hormone levels in postmenopausal women with Type 2 diabetes. Biomed. Pharmacother..

[70] Hunter D.C., Brown R., Green T., Thomson C., Skeaff M., Williams S., Todd J.M., Lister C.E., McGhie T., Zhang J. (2011). Changes in markers of inflammation, antioxidant capacity and oxidative stress in smokers following consumption of milk, and milk supplemented with fruit and vegetable extracts and vitamin C. Int. J. Food Sci. Nutr..

[71] Biniaz V., Tayebi A., Ebadi A., Shermeh M.S., Einollahi B. (2014). Effect of vitamin C supplementation on serum uric acid in patients undergoing hemodialysis: A randomized controlled trial. Iran. J. Kidney Dis..

[72] El Mashad G., Elsayed H., Nosair N. (2016). Effect of vitamin C supplementation on lipid profile, serum uric acid, and ascorbic acid in children on hemodialysis. Saudi J. Kidney Dis. Transplant..

[73] Choudhury M.R., Huq S., A Saleh A., Hakim F., Azad A.K. (2016). Efficacy of Vitamin C in Lowering Serum Uric Acid. Mymensingh Med. J..

[74] Peng H., Feng D., Wang Y., Dong Z., Chen Q., Zhang L., Luo R., Chen J., Wang A., Ma S. (2018). Effect of Oral Vitamin C Supplementation on High-Altitude Hyperuricemia in Young Men Initially Migrating to High Altitude: A Pilot Study. High Alt. Med. Biol..

[75] Stamp L.K., O’Donnell J.L., Frampton C., Drake J.M., Chapman P.T., Zhang M. (2013). Clinically Insignificant Effect of Supplemental Vitamin C on Serum Urate in Patients with Gout: A Pilot Randomized Controlled Trial. Arthritis Rheum..

[76] Azzeh F.S., Al-Hebshi A.H., Al-Essimii H.D., Alarjah M.A. (2017). Vitamin C supplementation and serum uric acid: A reaction to hyperuricemia and gout disease. PharmaNutrition.

[77] Brites F.D., Evelson P.A., Christiansen M.G., Nicol M.F., Basílico M.J., Wikinski R.W., Llesuy S.F. (1999). Soccer players under regular training show oxidative stress but an improved plasma antioxidant status. Clin. Sci..

[78] Juraschek S.P., Miller E.R., Gelber A.C. (2011). Effect of oral vitamin C supplementation on serum uric acid: A meta-analysis of randomized controlled trials. Arthritis Rheum..

